# Genetic diversity of *Chamaecrista fasciculata (Fabaceae)* from the USDA germplasm collection

**DOI:** 10.1186/s13104-019-4152-0

**Published:** 2019-03-04

**Authors:** Erika Bueno, Ted Kisha, Sonja L. Maki, Eric J. B. von Wettberg, Susan Singer

**Affiliations:** 1 Plant and Soil Science, University of Vermont, Burlington, VT USA; 2 USDA ARS, Pullman, WA USA; 3 Biology and Cognitive Science Department, Carleton College, Northfield, MN USA; 4 Plant and Earth Science Department, University of Wisconsin-River Falls, River Falls, WI USA; 5 Department of Biology, Rollins College, Winter Park, FL USA

**Keywords:** *Chamaecrista*, Caesalpinoid, Population genetics, ALFP markers, Genetic diversity

## Abstract

**Objective:**

*Chamaecrista fasciculata* is a widespread annual legume across Eastern North America, with potential as a restoration planting, biofuel crop, and genetic model for non-papillinoid legumes. As a non-Papilinoid, *C. fasciculata*, belongs to the Caesalpiniod group in which nodulation likely arose independently of the nodulation in Papilinoid and Mimosoid legumes. Thus, *C. fasciculata* is an attractive model system for legume evolution. In this study, we describe population structure and genetic diversity among 32 USDA germplasm accessions of *C. fasciculata* using 317 AFLP markers developed from 12 primer pairs, to assess where geographically there is the most genetic variation.

**Results:**

We found that the *C. fasciculata* germplasm collection fall into four clusters with admixture among them. After correcting for outliers, our analysis shows two primary groups across Eastern and Central North America. To better understand the population biology of this species, further sampling of the full range of this widespread species is needed across North America, as well as the development of a larger set of markers providing denser coverage of the genome. Further sampling will help clarify geographical relationships in this widespread temperate species.

**Electronic supplementary material:**

The online version of this article (10.1186/s13104-019-4152-0) contains supplementary material, which is available to authorized users.

## Introduction

Genetic diversity of germplasm collections serves as an important resource for the conservation and maintenance of both wild and cultivated plants and can be particularly useful for the development of new potential crops. One such species is *Chamaecrista fasciculata,* or partridge pea, which is a member of the economically important Leguminosae family. The species belongs to the subfamily Caesalpinioideae; the common ancestor of Papilionoid legumes (soybean, *Medicago*, and *Lotus*) which diverged approximately 60 million years ago (Legume Phylogeny Group, [20, 21] from these groups. There is growing interest in implementing *Chamaecrista* as a complementary model for legume evolution due to its relatively small genome size, phylogenetic position, ability to form nodules, and flower development; all of which would provide fundamental knowledge on the evolutionary origins of legume traits [29]. A genome sequencing project is currently underway for *C. fasciculata* (Steve Cannon, Pers. Comm.), which is one of the only annual temperate species with a compact growth form in the large genus of ~ 330 mostly long-lived tropical tree and shrub species.

The partridge pea (*C. fasciculata*), is a North American annual legume with a widespread distribution that ranges from the Northern Great Plains to Central Mexico. In the U.S. *C. fasciculata*, can be found growing from southern New England to Florida and westward into New Mexico and Oklahoma [15]. It is self-compatible and has a high outcrossing rate of 80% [10, 12]. The plant produces large yellow flowers that are exclusively pollinated by carpenter bees and bumblebees [1]. Seeds are dispersed short distances from parents (< 2.5 m) via explosive dehiscence [10]. Below ground, *C. fasciculata* forms nodules in response to nitrogen fixing bacteria known as rhizobium [22]. Unlike other legume crops, the genus *Chamaecrista* has not undergone any whole genome duplications [2] since its divergence from the Papilionoideae and has a generally smaller genomes *(*ca. 650 Mb in *C. fasciculata*). Working with fewer copies of genes in a model system such as *C. fasciculata* makes genetic approaches substantially easier, potentially enhancing the rate of discovery in legume crops. As the only temperate annual in a large tropical tree genus, a wealth of information exists on the ecology of *C. fasciculata* including the characterization of locally adaptive traits in response to climate change, key pollinators, and gene flow and genetic structure among naturally occurring populations [4–6, 10, 11, 13, 14, 30, 31]. Additionally, the genus *Chamaecrista* has independently evolved the ability to form nodules, thereby creating a unique opportunity to investigate the origins of nodulation and mutualistic interactions in Leguminosae [3]. Therefore, expanding on the genomics of *C. fasciculata* as a non-papilionid model legume is a key step into understanding the evolution of legume traits.

Here, we characterize genetic variation in the USDA collection of *C. fasciculata* comprising of 32 accessions originating from a range of populations in the U.S. that span its geographic distribution. Using Amplified Fragment Length Polymorphism (AFLP) markers [33], we show that there are four clusters in the germplasm collection with minimal genetic differentiation among groups.

## Main text

### Methods

#### Germplasm collection

Accessions were selected from the USDA GRIN repository. In total, we assembled a total of 32 accessions which is a representative of all available accessions in the repository. Because the samples were donated to USDA prior to 1992, they lack precise location information. Thus, we were only able to determine the U.S. state from which they originated. All samples were of *C. fasciculata* var *fasciculata*, as *C. fasciculata* var *macrospermum* is restricted to Virginia, a state with no samples in this dataset.

#### AFLP marker development

Freeze-dried, leaf tissue samples from 32 accessions were pulverized in a SPEX SamplePrep 2000 Geno/Grinder^®^, and DNA was extracted using the Wizard^®^ Magnetic 96 DNA Plant System (Promega). Amplified Fragment Length Polymorphism (AFLP) markers were generated using locally developed procedures based on technology by Vos et al. [33] and following modifications in Johnson et al. [18] and Greene et al. [16]. We performed a restriction double digest in 25 µl reactions containing 250 ng of DNA, 1X Purified BSA, 5.0 U each of *Eco*RI and MseI restriction enzymes (New England BioLabs) and 1X NE Buffer 4. To verify complete digestion, re ran 15 µl of the restriction digest reaction on a 1.5% agarose gel.

Adapter sequences (*Eco*RI-Fwd, 5′-ctc gta gac tgc gta cc; *Eco*RI-Rev, 5′-aat tgg tac gca gtc tac; MseI-Fwd, 5′-gac gat gag tcc tga g, and MseI-Rev, 5′-tac tca gga ctc at) were purchased from Eurofins MWG/Operon (Huntsville, Alabama). After diluting each adapter pair to 100 pM/µl (*Eco*RI) or 200 pM/µl (MseI), we combined them in equal amounts, and let them anneal for 1 h at 37 °C and cool to room temperature. We then diluted the annealed pairs to 5 pM/µl (*Eco*RI) and 50 pM/µl (MseI), aliquoted to 100 µl amounts for frozen storage for possible future use.

Following previous procedures in Johnson et al. [18] and Greene et al. [16], we performed a ligation step at 20° C for 2 h in a 20 µl reaction containing 10 µl of the remaining restriction digest, 5 pMoles *Eco*RI adapter, 50 pMoles MseI adapter, 0.5 mM ATP, 80 cohesive end Units of T4-ligase, and 1X T4 Ligase Buffer (New England BioLabs). We diluted the completed ligation reaction to 10:1 for pre-amplification. Both pre-amplification and selective amplification were done using an ABI 9700 thermocycler using cycling programs described by Vos et al. [33] in 10 µl reactions. Two millilitre of the diluted pre-amplification product (10:1) was used for selective amplification. We used twelve separate primer pairs for selective amplification (Eacg/Mcaa, Eagg/Mcaa, EacaMcag, EaccMcat, Eacg/Mctg, Eagc/Mctt, Eaca/Mcta, Eacc/Mctc, Eacg/Mcac, Eagg/Mctg, Eaca/Mcat, Eacc/Mcaa) where the last 3 letters indicate the selective nucleotides following the E-*Eco*RI and M_MseI primer sequences). Marker fragments were visualized on a LI-COR 4300 DNA Analyzer (LI-COR Biosciences). We scored marker loci as either present or absent based on printed images.

#### Data analysis

We created a graphical display of accession relationships with NTSys-pc software [27] using Jacard’s coefficient. The tree was constructed using Q-values that were outputted from a STRUCTURE analysis (see below) at K = 4 and Prevosti’s distance coefficient [25] which substitutes Q-value fractions for allele frequencies at a single AFLP locus.

To examine population structure we used STRUCTURE v2.3.3 [8, 9, 26] and the widely applied technique developed by Evanno et al. [7]. Ten replications with a burn-in of 20,000 iterations followed by 20,000 additional iterations were used at each K level until results indicated lowered and less erratic values for P(X|K). The parameter set included the ADMIXTURE model with allele frequencies correlated, and a RECESSIVE ALLELES model that is essential for dominant loci like AFLPs. Average Q-plots over the ten replications were calculated using the associated software CLUMPP [17], and graphic displays of population structure were developed from the q-frequencies of the mean of 10 runs using DISTRUCT software [28]. We analyzed genetic diversity in Genalex 6.5 [23, 24] and checked them in AFLP-SURV 1.1 [32] (not shown). Lastly, we performed a Principal Components Analysis (PCA) for clustering using binary assignments in Genalex.

### Results

#### Analyses of population structure

**Fig. 1:**
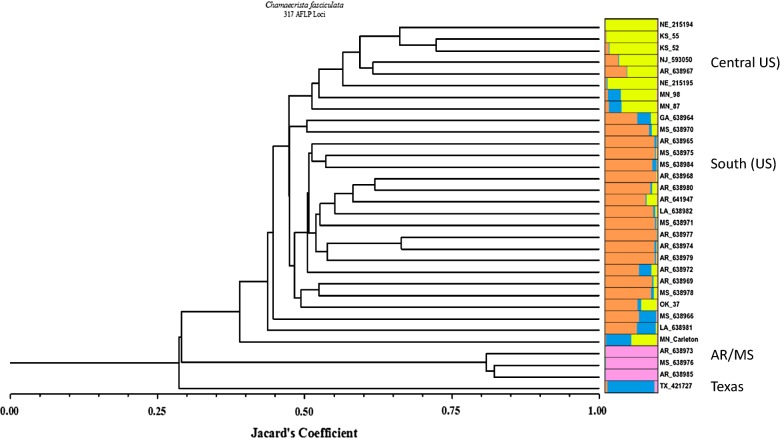
Phenogram of 32 *Chamaecrista fasciculata* accessions from 317 AFLP loci using Jacard’s Coefficient. Results of STRUCTURE analysis at K = 4 superimposed on the phyogenetic tree using DISTRUCT software. Each STRUCTURE group is represented by a different color, which mixed colors for individuals indicating admixture. We define the groups as Central 1 (yellow), South 2 (orange), AR-MS (for Mississippi and Arkansas, pink) and Texas (blue). The two letters after each accession indicate the US state from which it originates

**Fig. 2:**
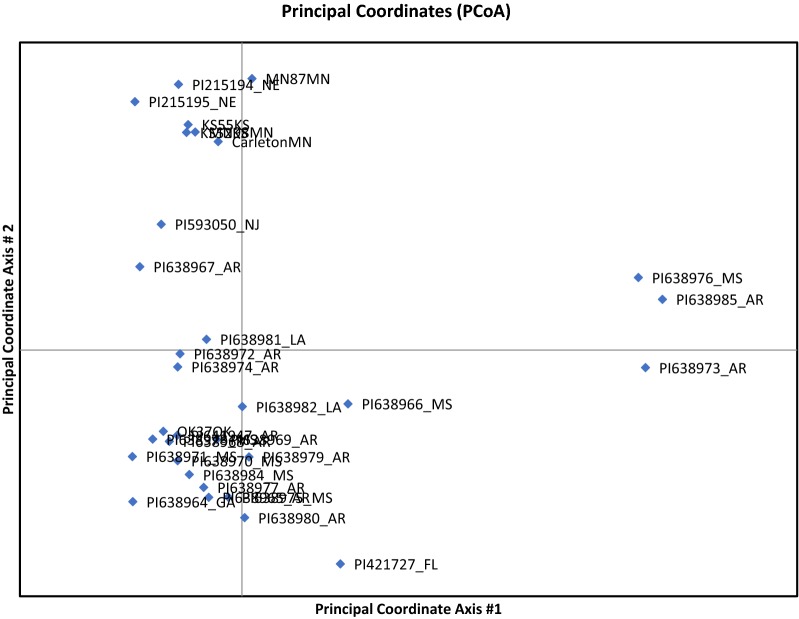
PCoA plot of 32 USDA *Chamaeacrista fasiculata* accessions. Three accessions from the US states of Mississippi (MS) and Arkansas (AR) form a group (MS-AR) that was also detected in our STRUCTURE analysis (Fig. 1). Accessions are named by USDA GRIN ID number and the US state from which they originate

AFLP analysis resulted in a total of 317 polymorphic loci. STRUCTURE analysis combined with the technique of Evanno et al. [7] indicated the most probable number of distinct populations at K = 4 (Figs. 1 and 2, Table 1, Additional file 1: Figure S1a, b). Separation was, for the most part, based on latitude with some anomalies. Consequently, we named these groups Central (US), South, AK/MS, and Texas. While the accessions from Kansas, Nebraska, New Jersey, and Minnesota (Central US group) were mostly separated from those of Arkansas and Mississippi, two accessions from Arkansas, and one from Mississippi were grouped apart from the others, and then placed into our AR/MS cluster. A sample from Texas also formed a separate group, although some samples from other states, such as Minnesota, showed some admixture with this group.

**Table 1:** Group assignments, based on STRUCTURE output analyzed in DISTRUCT

Name	Geographic location	Assigned group	AvgG1: Central US	AvgG2 South US	AvgG3 AR/MS	AvgG4 TX
MN87MN	Minnesota	1	0.84766	0.10144	0.00464	0.04622
PI638984_MS	Mississippi	1	0.902	0.0106	0.00286	0.0845
PI638972_AR	Arkansas	1	0.73666	0.10594	0.00338	0.15402
CarletonMN	Minnesota	1	0.90492	0.0676	0.00538	0.02212
PI421727_FL	Florida	1	0.78614	0.00676	0.06742	0.13968
PI215194_NE	Nebraska	2	0.0045	0.9867	0.00254	0.00628
KS55KS	Kansas	2	0.0062	0.98176	0.00414	0.00792
KS52KS	Kansas	2	0.01128	0.97022	0.00372	0.01476
PI593050_NJ	New Jersey	2	0.06028	0.7166	0.00362	0.21948
PI215195_NE	Nebraska	2	0.016	0.95024	0.00234	0.03138
MN98MN	Minnesota	2	0.19864	0.7373	0.02154	0.0425
PI638973_AR	Arkansas	3	0.00358	0.0024	0.99056	0.00342
PI638976_MS	Mississippi	3	0.00352	0.00656	0.98712	0.00278
PI638985_AR	Arkansas	3	0.00202	0.00226	0.99362	0.0021
PI638964_GA	Georgia	4	0.0106	0.01294	0.00222	0.97424
PI638970_MS	Mississippi	4	0.04758	0.00958	0.00418	0.9387
PI638965_AR	Arkansas	4	0.28276	0.01834	0.0052	0.69372
PI638968_AR	Arkansas	4	0.01312	0.04472	0.00324	0.93894
PI641947_AR	Arkansas	4	0.03324	0.04714	0.00398	0.91562
PI638971_MS	Mississippi	4	0.2241	0.01648	0.00258	0.75686
PI638977_AR	Arkansas	4	0.01602	0.00734	0.00304	0.97358
PI638974_AR	Arkansas	4	0.03314	0.00978	0.00808	0.94906
PI638979_AR	Arkansas	4	0.08862	0.02068	0.0084	0.88232
PI638978_MS	Mississippi	4	0.08274	0.02068	0.00266	0.89388
OK37OK	Oklahoma	4	0.08768	0.02706	0.00282	0.8824
PI638967_AR	Arkansas	Admixed	0.02898	0.46856	0.00282	0.49962
PI638975_MS	Mississippi	Admixed	0.56434	0.01114	0.01008	0.41442
PI638980_AR	Arkansas	Admixed	0.53272	0.03362	0.00578	0.42788
PI638982_LA	Louisiana	Admixed	0.50036	0.0193	0.0153	0.46506
PI638969_AR	Arkansas	Admixed	0.34768	0.02832	0.0079	0.61608
PI638966_MS	Mississippi	Admixed	0.48354	0.01372	0.20876	0.29398
PI638981_LA	Louisiana	Admixed	0.60256	0.10074	0.00492	0.29174

Our STRUCTURE analysis detected four groups, or populations, which we have named Central (group 1, yellow in Fig. 1), South (group 2, orange in Fig. 1), AR/MS (group 3, Arkansas/Mississippi, pink), and Texas (TX, group 4, blue). We give the percent membership of each accession to each STRUCTURE group to show the extent of admixture

We identified seven individuals as considerably admixed among at least two of the groups. A Principal Component Analysis (PCA, Fig. 2) showed the three individuals from the AR-MS group differentiated on the first axis, and differentiation along a latitudinal axis on the second axis. Although STRUCTURE combined the more Northern accessions to the first two groups (our Central and South groups), the PCA suggests a subtle latitudinal cline in diversity, overwhelmed by differentiation among multiple groups in the Southern US. This pattern of greater Southern diversity and differentiation is consistent with glacial refugia in the Southern U.S. during the last glacial maxima, and admixture as populations migrated back to deglaciated areas in the more Northern US.

#### Genetic diversity analysis

Overall, we found some genetic differentiation among the four groups in the USDA *Chamaecrista fasciculata* germplasm collection. In total, we analyzed the genetic variability of 317 loci from 32 *C. fasciculata* accessions (Table 2). The overall Pairwise genetic distance PhiPT value was 0.207 (P = 0.001). The Analysis of Molecular Variance (AMOVA) based on PhiPT values indicated that 79% of the variance comes from within populations (estimated variance = 11.84) while 21% of the variance comes from among populations (estimated variance = 3.11). Mean Shannon’s diversity index across all populations was 0.24 (± 0.11).

**Table 2:** Genetic diversity in 317 AFLP loci in 32 USDA accessions of *Chamaecrista fasciculata*

	N	Na	Ne	I	H	Uh	%P
Pop							
Central US							
Mean	5.000	1.297	1.402	0.355	0.239	0.298	62.71
SE	0.000	0.086	0.033	0.026	0.018	0.022	
South (US)							
Mean	6.000	0.932	1.236	0.209	0.140	0.168	38.14
SE	0.000	0.084	0.031	0.025	0.017	0.021	
AR/MS							
Mean	3.000	0.466	1.081	0.065	0.045	0.068	10.17
SE	0.000	0.062	0.022	0.018	0.012	0.019	
Texas							
Mean	11.000	1.263	1.264	0.267	0.169	0.186	59.32
SE	0.000	0.085	0.028	0.023	0.016	0.017	
Admixed							
Mean	7.000	1.246	1.332	0.306	0.201	0.235	58.47
SE	0.000	0.086	0.032	0.025	0.017	0.020	
Grand mean and SE over loci and pops							
Total							
Mean	6.400	1.041	1.263	0.240	0.159	0.191	45.76
SE	0.109	0.038	0.014	0.011	0.008	0.009	9.89

Na = no. of different alleles, Ne = no. of effective alleles = 1/(p^2 + q^2), I = Shannon’s Information Index = −1* (p * Ln (p) + q * Ln(q)), H = diversity = 1 − (p^2 + q^2), uh = unbiased diversity = (N/(N−1)) * h (where for haploid binary data, p = Band Freq. and q = 1 − p), and  %P = percent polymorphic loci

### Discussion

AFLP markers were used to estimate genetic diversity among 32 *C. fasciculata* accessions sampled across its geographical distribution. The patterns of differentiation we observed in *C. fasciculata* likely result in part from migration in response to repeated patterns of glacial activity. The differentiation found in the more Southern US states is likely a result of differentiation in glacial refugia, such as on different sides of the Appalachian mountain chain or Ozark mountains, with more Northern populations resulting from post-glacial advances northward and possible admixture from different glacial refugia. A similar AFLP analysis of *Phaseolus polystachios*, the North American Wild Kidney Bean, and the only *Phaseolus* species native to temperate North America set apart an accession from Texas which was later given species status as *Phaseolus texensis* ([19], and unpublished).

*Chamaecrista fasciculata* is a very widespread plant in eastern and central North America, occurring in a variety of habitats from mixed prairies to disturbed habitats, to unique local ecosystems such as mid-Atlantic serpentine barrens and South Florida Karstic pine rocklands. Such widespread occurrence and broad adaptation could make it useful as a component of mixed biofuel plantings as well as habitat restoration plantings and ecological and evolutionary studies. Based on our findings, the current collection, although diverse, likely does not capture the full range of variation present in this ecologically diverse species. In particular, more precise sampling from particular habitats, may show unique patterns of differentiation. Similarly, more thorough sampling at the edge of the geographic range of the species may find outlying populations, or uncover introgression with more tropical *Chamaecrista* species, such as *C. nictitans* or *C. lineata* var. *keyensis,* which is endangered in the Florida Keys. The outlying Texas group may be consistent with range-edge differentiation of populations. Thus, we recommend further collecting to improve the value of this collection for a variety of uses, from research to restoration, to biofuels.

## Limitations

The AFLP markers that were used in this study have several limitations such as being dominant rather than co-dominant, occurring at random locations in the genome that are difficult to tie to a genomic region and being limited to a few hundred total loci. New technologies, such as genotyping-by-sequencing and next generation sequencing based approaches that develop single nucleotide polymorphisms do overcome these challenges. Secondly, the set of lines examined is small in total number, with 32 being marginal for inference about population genetic patterns. Third, the USDA collection was assembled before 1992, when GPS units became available. Consequently, the passport data for the accessions we assessed is limited to U.S. State, rather than more precise locations. Our work suggests that efforts to expand the USDA germplasm collection for *Chamaecrista* and improve the associated passport data would be quite useful for a number of research applications.

## Additional file

**Additional file 1: Figure S1.** Plots from the software STRUCTURE of A) lnP(X|K) indicating the highest probability at K = 4, and (B) graph of dK vs K from technique of Evanno et al. [7] indicating most probable population subdivisions at K = 2 and K = 4. Based on the Evanno et al [7] technique, we find 4 to be the best number of populations.
