# Supplementary figures and images for: Genetic diversity of Chamaecrista fasciculata (Fabaceae) from the USDA germplasm collection
Source: BMC Res Notes. 2019 Mar 4;12:117. doi: 10.1186/s13104-019-4152-0 (PMC6400026; doi:10.1186/s13104-019-4152-0)

## Slide 1
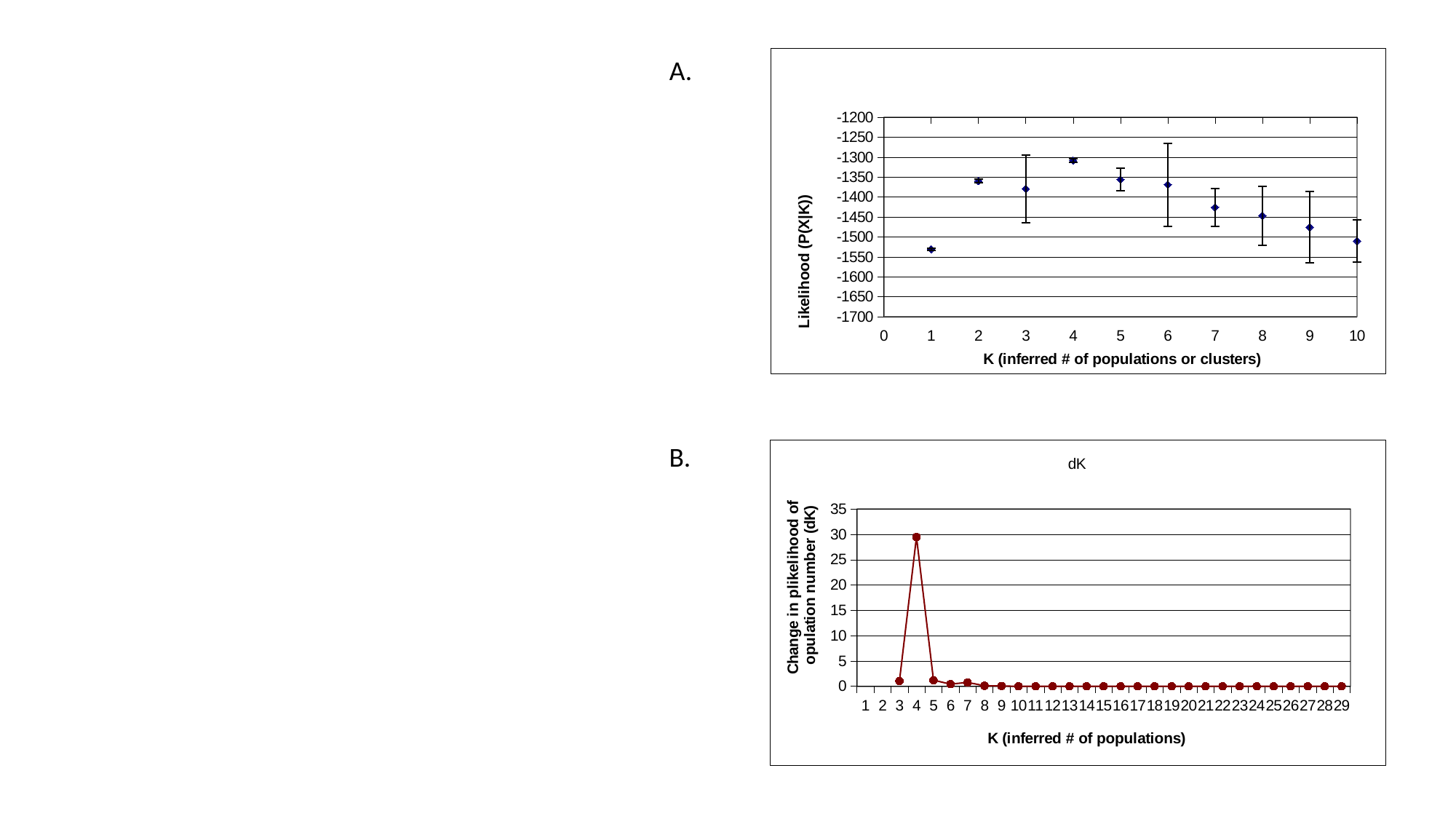

A.
### Chart
| Category | L(K) |
|---|---|B.
### Chart:
| Category | dK |
|---|---|
| 1 | None |
| 2 | None |
| 3 | 1.0584299457976234 |
| 4 | 29.494195014602592 |
| 5 | 1.206040576082133 |
| 6 | 0.42388023445387973 |
| 7 | 0.7648952676471353 |
| 8 | 0.11190901828152484 |
| 9 | 0.060685432004713415 |
| 10 | 0.0 |
| 11 | 0.0 |
| 12 | 0.0 |
| 13 | 0.0 |
| 14 | 0.0 |
| 15 | 0.0 |
| 16 | 0.0 |
| 17 | 0.0 |
| 18 | 0.0 |
| 19 | 0.0 |
| 20 | 0.0 |
| 21 | 0.0 |
| 22 | 0.0 |
| 23 | 0.0 |
| 24 | 0.0 |
| 25 | 0.0 |
| 26 | 0.0 |
| 27 | 0.0 |
| 28 | 0.0 |
| 29 | 0.0 |

Supplement: Supplementary file 1 — Additional file 1: Figure S1. Plots from the software STRUCTURE of A) lnP(X|K) indicating the highest probability at K = 4, and (B) graph of dK vs K from technique of Evanno et al. [7] indicating most probable population subdivisions at K = 2 and K = 4. Based on the Evanno et al [7] technique, we find 4 to be the best number of populations. [file 13104_2019_4152_MOESM1_ESM.pptx]
